# Analysis of miRNA-mediated regulation of flowering induction in *Lilium* × *formolongi*

**DOI:** 10.1186/s12870-021-02961-3

**Published:** 2021-04-20

**Authors:** Qian Zhang, Yu-Qian Zhao, Xue Gao, Gui-Xia Jia

**Affiliations:** Beijing Key Laboratory of Ornamental Plants Germplasm Innovation and Molecular Breeding, National Engineering Research Center for Floriculture, Beijing Laboratory of Urban and Rural Ecological Environment and College of Landscape Architecture, Beijing Forestry University, Beijing, China

**Keywords:** *Lilium* × *formolongi*, Development, Flowering, miRNA

## Abstract

**Background:**

MicroRNAs play pivotal roles in plant vegetative phase change and flowering induction via integrating into multiple flowering pathways. *Lilium* × *formolongi* is an important ornamental lily cultivar that can flower within one year after sowing. However, it remains unresolved how miRNA-mediated regulation networks contribute to the *L.* × *formolongi* characteristics of a short vegetative growth period and rapid flowering.

**Results:**

In this study, the small RNA libraries and one degradome library were constructed for *L.* × *formolongi* during vegetative growth and flowering initiation, and 366 conserved miRNAs and 32 novel miRNAs were identified. Additionally, 84 miRNAs were significantly differentially expressed during development. A total of 396 targets of 185 miRNAs were identified and validated through degradome sequencing. Gene Ontology (GO) and Kyoto Encyclopedia of Genes and Genomes (KEGG) pathway analyses showed that functions of the targets were top enriched in the cold and cadmium ion responses, pentose phosphate pathway and carbon fixation in photosynthetic organisms. Furthermore, among 23 differentially expressed miRNA-target pairs, the miR156s-*LfSPL2*, miR172a-*LfAP2* and miR164a-*LfNAC* pairs as well as miR159a-*LfSPL2* were found to be relevant to flowering based on the correlation analysis of expression profiles in the miRNA libraries, degradome and transcriptome. A coexpression regulatory network focused on differentially expressed pairs was also constructed by WGCNA, and 14 miRNAs were considered putative key miRNAs during vegetative development and flowering induction. miR156a/ d/ e showed particularly strong relationships with other miRNAs in the coexpression network.

**Conclusions:**

This study provides cues for the further exploration of the regulatory mechanisms of short vegetative development and flowering in *L.* × *formolongi*.

**Supplementary Information:**

The online version contains supplementary material available at 10.1186/s12870-021-02961-3.

## Background

*Lilium* (Liliaceae family) is an important genus including crops that are valued for their ornamental characteristics, edible bulbs and medical use. They can be propagated by either sexual or asexual propagation. Sexual propagation through seed reproduction enables breeders to obtain virus-free seedlings and maintain abundant genetic diversity [1]. In most lily cultivars and wild species, at least 1 year of vegetative growth is required for flowering after sowing [2]. However, *L.* × *formolongi*, a popular cut flower cultivar with the characteristic of a shortened vegetative phase, can undergo floral transition within one year after germination [3]. Therefore, the investigation of molecular pathways involved in *L.* × *formolongi* vegetative development and flowering initiation is necessary to shorten the vegetative growth period and advance the flowering time of lily.

Vegetative growth after germination is necessary for angiosperm plants to achieve competence to flower and respond to external stimuli after the juvenile-adult stage transition, followed by the transition to reproductive growth [4]. As such, the optimal timing of the transition from the vegetative to the reproductive phase and flowering is precisely controlled by a complex genetic network composed of multiple pathways. In the annual plant *Arabidopsis thaliana*, external factors such as day length and temperature affect the photoperiod and vernalization pathways [5]. They control phase transitions and flowering time in coordination with ageing, gibberellic acid (GA), trehalose 6-phosphate (T6P) and autonomous pathways governed by endogenous factors including age, hormones and carbohydrate status [5, 6]. Currently, a large number of studies suggest that miRNAs are also integrated into flowering pathways, in which they perform orchestrated functions [7].

Mature plant miRNAs exert regulatory effects primarily by directly cleaving mRNAs or repressing the translation of specific mRNAs [8, 9]. In *Arabidopsis*, several endogenous conserved miRNAs and their targets are integrated into the ageing pathway, in which they influence development phase changes and flowering time [10, 11]. miR156, the orchestrator of the pathway, plays a master regulatory role in vegetative development via transcript cleavage or the translational repression of target *SQUAMOSA PROMOTER BINDING PROTEIN-LIKE* (*SPL*/ *SBP*) genes, resulting in the transition to the adult stage [12, 13]. Conversely, miR172 targets six *APETALA 2* (*AP2*)-*like* genes that function in flowering repression, and the abundance of miR172 increases with age [14, 15]. In apices, miR172 acts downstream of the miR156/ *SPL* module, and *SPL9* and *SPL15* can alter the expression of miR172 by directly binding to the miR172 precursor *MIR172b* [16]. In addition to the two components of the ageing pathway, the examination of the phenotypes of mutant and transgenic plants has demonstrated that miR159, which is repressed by the DELLA protein in the GA pathway, is involved in the vegetative phase transition, independent of GA, and represses the expression of miR156 [17, 18]. In tomato, miR156-*SlSBPs* interact with GA and the miR319-*LANCEOLATE* (*LA*) module to affect flowering induction and the floral transition [19]. Additionally, overexpressed miR171 can activate miR156 to influence the vegetative-to-reproductive phase change in barley [20].

Many species-specific or less conserved miRNAs have also been identified and further confirmed to be involved in flowering induction in plants. Monocot-specific miRNA miR528 has been found to promote rice flowering induction under long-day conditions [21]. Brassicaceae-specific miRNA miR824 and its target *AGAMOUS-LIKE* 16 (*AGL16*) modulate flowering time by repressing the expression of *FT* under long-day conditions in *Arabidopsis* [22]. *Pooideae*-specific miR5200 has been shown to regulate the photoperiod-mediated flowering transition by guiding the sequence-specific cleavage of *FT* orthologues in *Pooideae* plants [23].

As a geophyte with underground storage organs, previous studies have confirmed that the flowering time of lily can be affected by multiple factors, including vernalization, photoperiod and bulb size [24, 25]. However, the molecular regulatory factors underlying the vegetative phase transition and responses to various floral induction signals in lily are still unknown. On the other hand, the lily genome is large, complex and lacks a reference genome sequence [26]. Nevertheless, sequencing technologies make it relatively easy to produce sufficient data for the identification of genes related to important agronomic traits, and this research could benefit from computational and degradome analyses that detect the targets of miRNAs rapidly and accurately [27, 28].

Therefore, 15 miRNA libraries and one degradome library were constructed for *L.* × *formolongi* at five key developmental stages. Databases, in conjunction with transcriptomic data, were further used to systematically identify and profile miRNAs and their targets during vegetative development and flowering initiation. Potential crucial flowering miRNA-target pairs were also identified. These results may contribute to elucidating the regulatory mechanism of miRNA-mediated short vegetative development in *L.* × *formolongi* and accelerating lily flowering.

## Results

### High-throughput sRNA sequencing in *L.* × *formolongi*

**Fig. 1:**
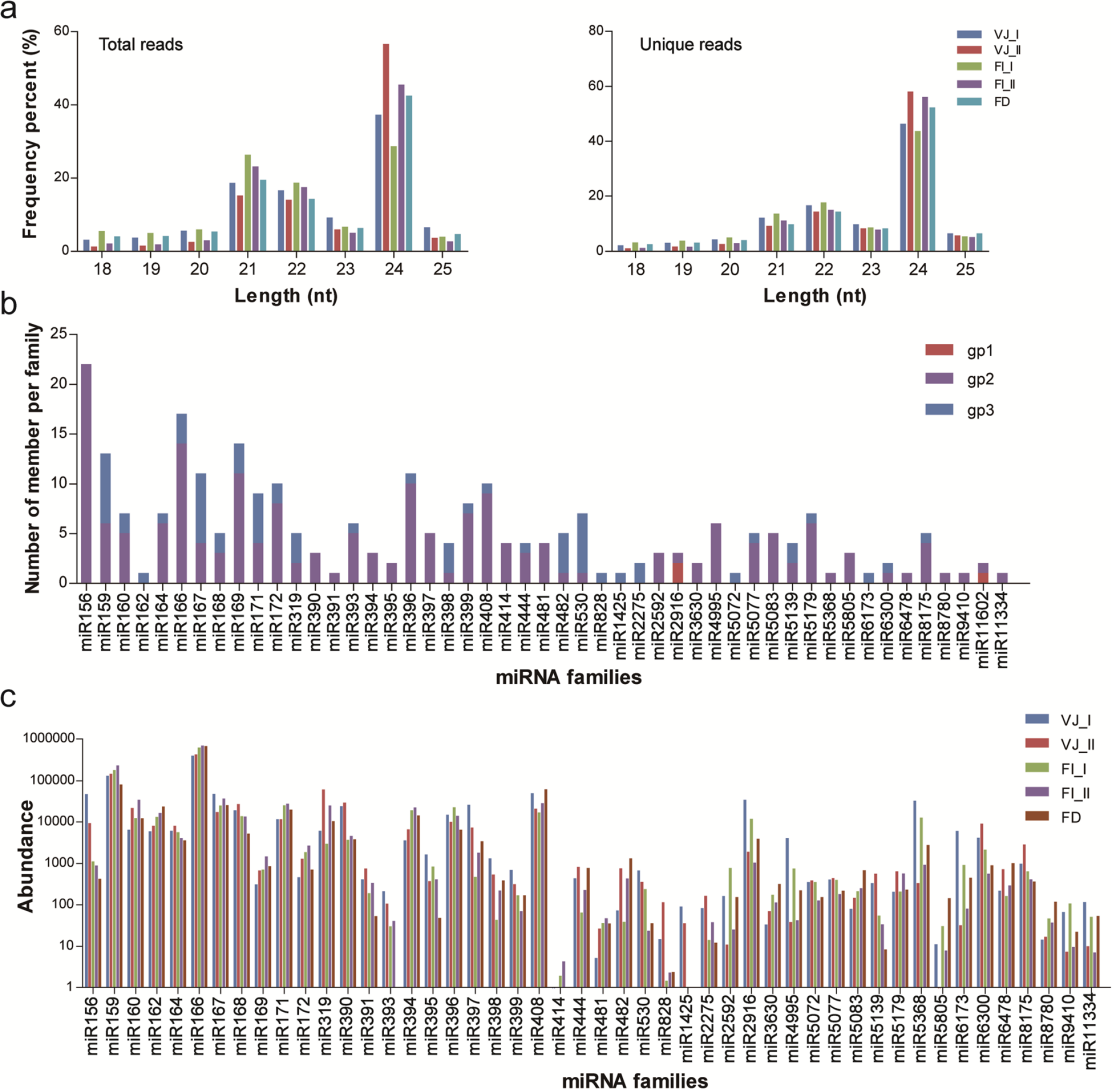
Length distribution of small RNAs sequences, identification of known miRNA families from *L.* × *formolongi*. **a** Length distribution of total reads and unique reads in five libraries. **b** Distribution of known miRNA family members. **c** Counts of each known miRNA family

To explore comprehensive miRNA data during *L.* × *formolongi* development, 15 small RNA libraries were constructed and sequenced from leaf and stem meristem tissues collected at five developmental stages (VJ_I, VJ_II, FI_I, FI_II and FD) (Additional file 1, Fig. S1, S2). In total, 87,720,826 raw reads were generated from these sRNA libraries (Table 1). After removing junk sequences, poor-quality reads and noncoding RNAs, approximately 9.9 million, 10.8 million, 14.7 million, 19.3 million and 5.6 million valid reads that exactly matched the precursors deposited in miRBase 22.0 were obtained from the VJ_I, VJ_II, FI_I, FI_II and FD libraries, respectively. These miRNAs showed unique size distributions. The length distribution of 60,351,351 raw reads and 14,126,406 unique reads was centred on the 21–24 nt range. The most frequent length of valid reads among the libraries was 24 nt, followed by 21 nt for total reads and 22 nt for unique reads (Fig. 1a).

**Table 1:** High-throughput sequencing statistics of *L.* × *formolongi* sRNAs

Category		VJ_I		VJ_II		FI_I		FI_II		FD	
		Total	unique	Total	unique	Total	unique	Total	unique	Total	unique
Raw reads		14,240,103	4,165,620	13,012,678	5,070,705	25,100,402	4,417,437	24,430,380	5,493,407	10,937,263	2,363,859
3ADT & length filter		3,253,881	1,696,297	1,726,013	1,370,377	8,960,668	1,903,704	4,503,056	1,379,069	5,065,486	855,271
Junk reads		43,445	18,415	44,301	25,086	55,442c	15,965	59,104	21,952	31,103	10,933
Rfam	rRNA	625,844	17,010	103,024	7038	830,461	18,275	152,864	8762	135,278	6417
	tRNA	334,519	3726	271,546	3448	429,462	3870	364,860	3249	74,368	1669c
	snoRNA	4261	380	5215	436	20,391	926	19,562	890	17,995	843
	snRNA	4524	430	4826	523	12,468	951	8310	761	5221	489
	others	35,921	1242	8421	678	97,281	1808	11,959	1010	14,357	683
	Total	1,005,069	22,788	393,032	12,123	1,390,063	25,830	557,554	14,672	247,219	10,101
Repeats		8622	451	8427	458	19,758	680	10,301	708	3934	323
valid reads		9,932,250	2,427,801	10,843,854	3,662,791	14,681,011	2,471,404	19,303,311	4,077,107	5,590,925	1,487,303

### Identification of conserved miRNAs in *L.* × *formolongi*

The valid reads from all libraries were subjected to mapping with the precursors deposited in miRBase 22.1 and the transcriptome of *L.* × *formolongi* to identify conserved miRNAs. A total of 345 conserved miRNA precursors and 366 unique miRNAs belonging to 116 miRNA families were discovered, and transcriptome localization was obtained for a majority of these miRNAs (Fig. 1b, Additional file 2: Table S1). In addition, the number of homologs was counted and varied among the different miRNA families. The largest family was the miR156 family, with 22 members, followed by the miR166 and miR169 families, which had 17 and 14 members, respectively. However, miR162, miR391, miR828 and miR5072 were each represented by only one member. Furthermore, two miR2916 members, one miR5523 member and one miR11602 member were identified as known miRNAs (group 1) whose reads and precursors could be perfectly mapped to the reported *Lilium* miRNAs/ pre-miRNAs sequences in miRBase and the transcriptome.

The relative abundance of these conserved miRNAs during lily development was characterized. As shown in Fig. 1c, the miRNAs exhibited remarkably different levels of abundance. The top read counts of miRNAs were obtained for the miR166 family, followed by the miR159 and miR408 families during developmental stages.

### Identification of novel miRNAs in *L*. × *formolongi*

The unannotated reads that were mapped to the transcriptome of *L*. × *formolongi* were filtered for novel miRNA prediction according to the criteria for the hairpin structure of the precursor. Collectively, 32 novel miRNAs were found among all miRNA libraries (Additional file 2: Table S2). The lengths of these mature novel miRNAs and the hairpin lengths of the novel pre-miRNAs were between 19 and 25 nt and 55 and 191 nt, respectively. The majority of the miRNAs in these intervals were 21 nt and 164 nt long, respectively. The free energy of these secondary structures required to maintain stable hairpin structures ranged from − 125.5 to − 20.9 kCal/ mol. Precursors forming stem loop structures among the novel miRNAs, such as miRn5, miRn8 and miRn10, were predicted and are presented in Additional file 3: Fig. S3.

### Differentially expressed miRNAs during development

**Fig. 2:**
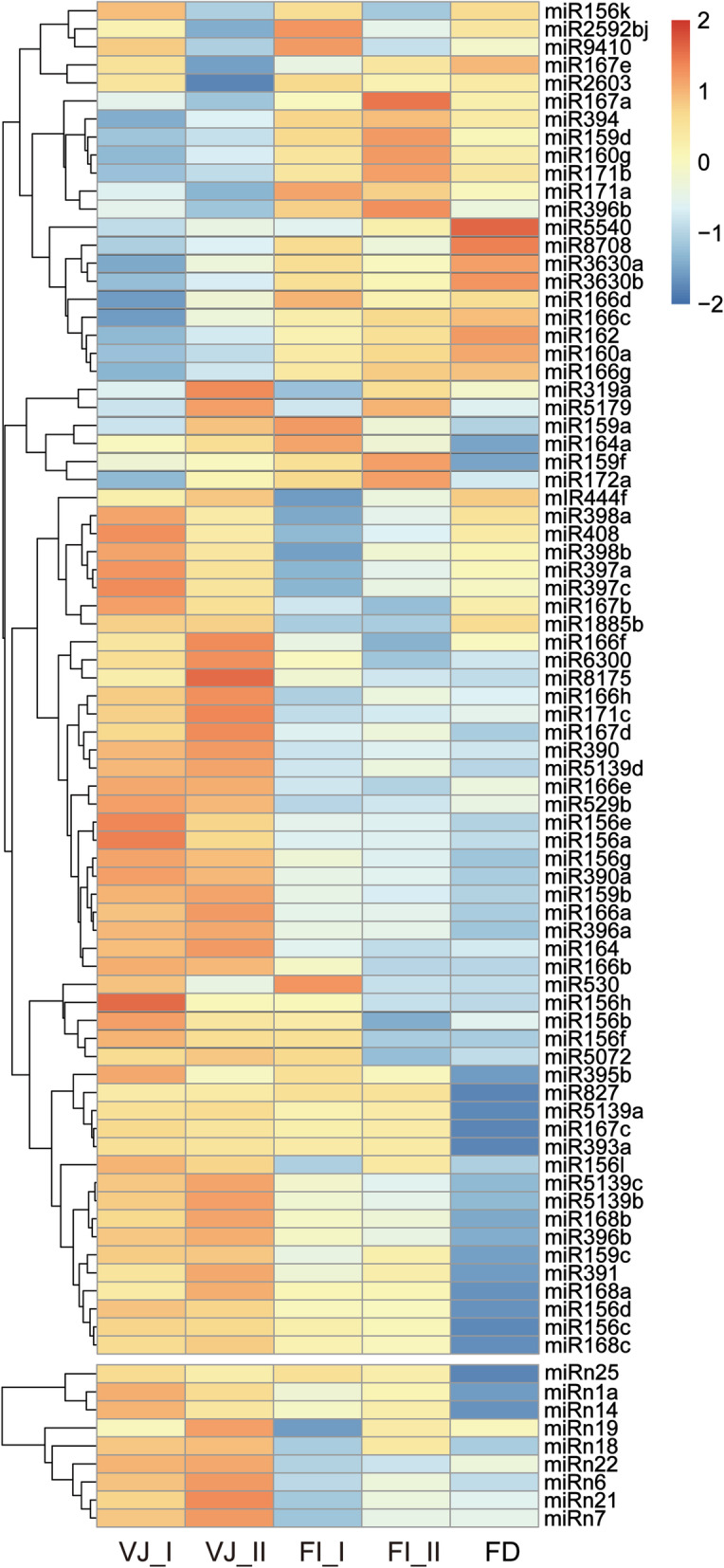
Heat map showing the expression patterns of differentially expressed miRNAs. The miRNAs were clustered by hierarchical clustering at *p* ≤ 0.05 according to their expression patterns during development in *L.* × *formolongi*

To identify the key miRNAs playing regulatory roles during vegetative growth and flowering initiation in lily, 75 conserved miRNAs and 9 novel miRNAs were identified as showing significant differences (Additional file 2: Table S3). Cluster analysis of their expression patterns was performed, and a heat map showing their distinct expression profiles was generated (Fig. 2). These miRNAs were characterized by three expression profiles with different expression changes from the VJ_ I to FI_II stages before FD.

The majority of miRNAs, including 50 conserved miRNAs and 9 novel miRNAs, presented high expression levels at the VJ (VJ_I and VJ_II) stage and were downregulated during later stages of development. In the miR156 family, most miR156s displayed consistently decreased expression, with the highest expression levels at the VJ_I or VJ_II stage. Similar to miR156s, most miR166 families exhibited expression peaks at the VJ_II stage. By contrast, 20 conserved miRNAs exhibited elevated expression from the VJ to FI_II stage or FD stage. miR159d, miR159f, miR172a and miR171 homologs exhibited upregulated expression levels from VJ_I to FI_II, and several less conserved miRNAs, such as miR5540, miR8708 and miR3630a/ b, maintained increased expression from the VJ to FD stages. In addition, many differentially expressed miRNAs with fluctuating transcription levels were found in the libraries. The expression of miR156k, miR2592bj and miR167e/ a was downregulated at the VJ_II and FI_II stages.

### Degradome sequencing

**Fig. 3:**
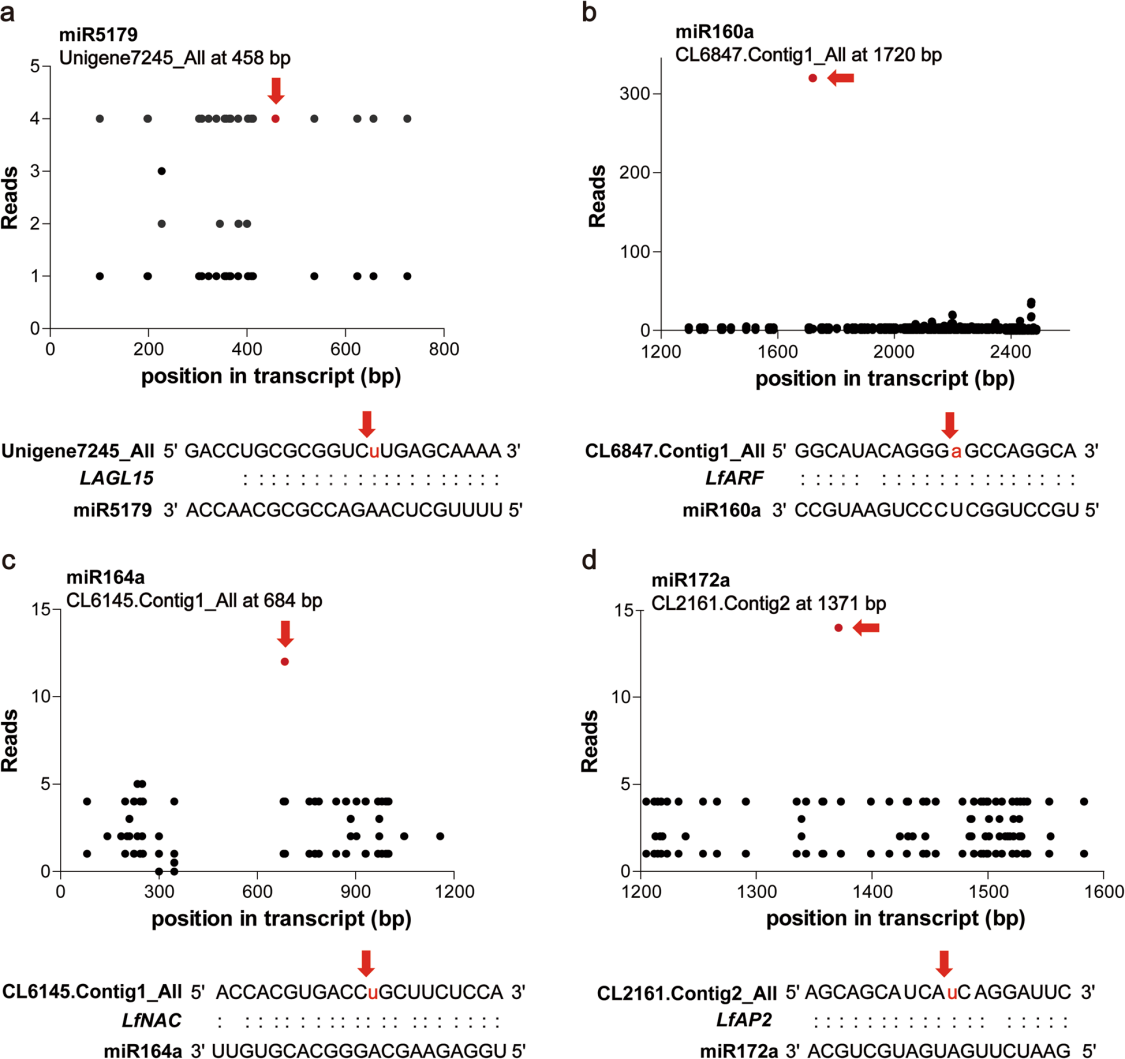
Target plots of miRNA targets identified by degradome sequencing in lily. **a**-**d** represent miR5179, miR160a, miR164a and miR172a, respectively. Arrows represent the nucleotide position of cleavage in the target genes

By combining the results of degradome and computational target prediction for the annotated corresponding mRNAs, 396 target transcripts of 235 miRNAs were globally identified in the degradome and analysed by detecting mRNA fragments that were diagnostic of miRNA-directed cleavage, including 157 conserved miRNAs (Fig. 3, Additional file 2: Table S4) and 28 novel miRNAs (Additional file 2: Table S5). According to the cleavage signature abundance at each occupied transcript position, we classified the identified degradome sequences into 5 categories based on the criteria indicated in previous studies [29] (Additional File 2: Table S4). Among these identified targets, 24, 7, 179, 25 and 184 belonged to categories 0, 1, 2, 3 and 4, respectively.

**Fig. 4:**
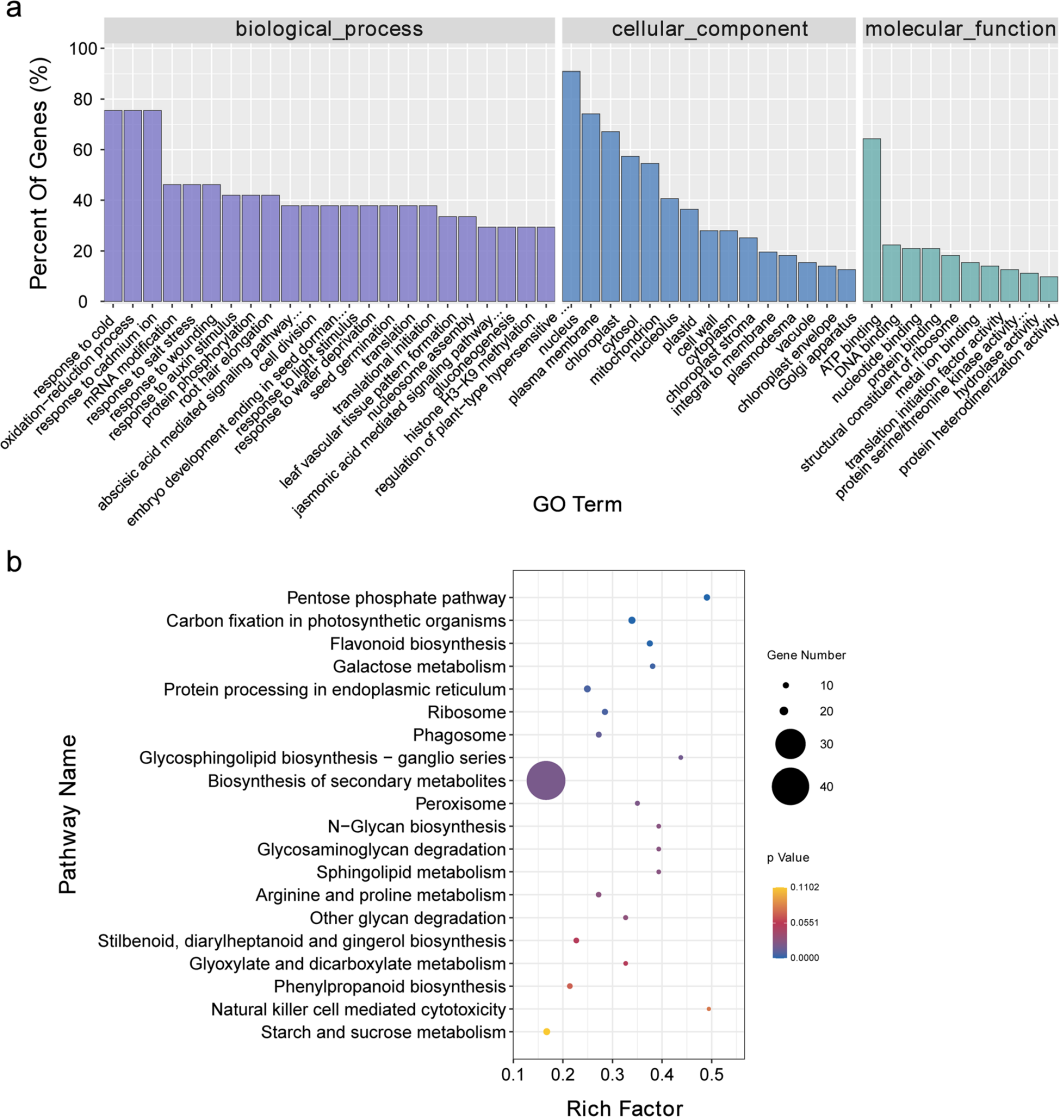
Gene functional classification of identified target genes. **a** Gene ontology enrichment and classification of target genes. **b** KEGG pathway analysis

The enriched GO items were analysed to elucidate the functions of the identified targeted genes. Among biological processes, ‘oxidation-reduction process’ (GO: 0055114), ‘response to cold’ (GO: 0009409) and ‘response to cadmium ion’ (GO: 0046686) were the most abundant categories. ‘Nucleus’ (GO: 0005634) and ‘ATP binding’ (GO: 0005524) were the most highly represented cellular component and molecular function categories, respectively (Fig. 4a). The enriched KEGG pathway analyses showed that the pentose phosphate pathway (ko00030), carbon fixation in photosynthetic organisms (ko00710) and flavonoid biosynthesis (ko00941) were the most enriched pathways (Fig. 4b).

**Fig. 5:**
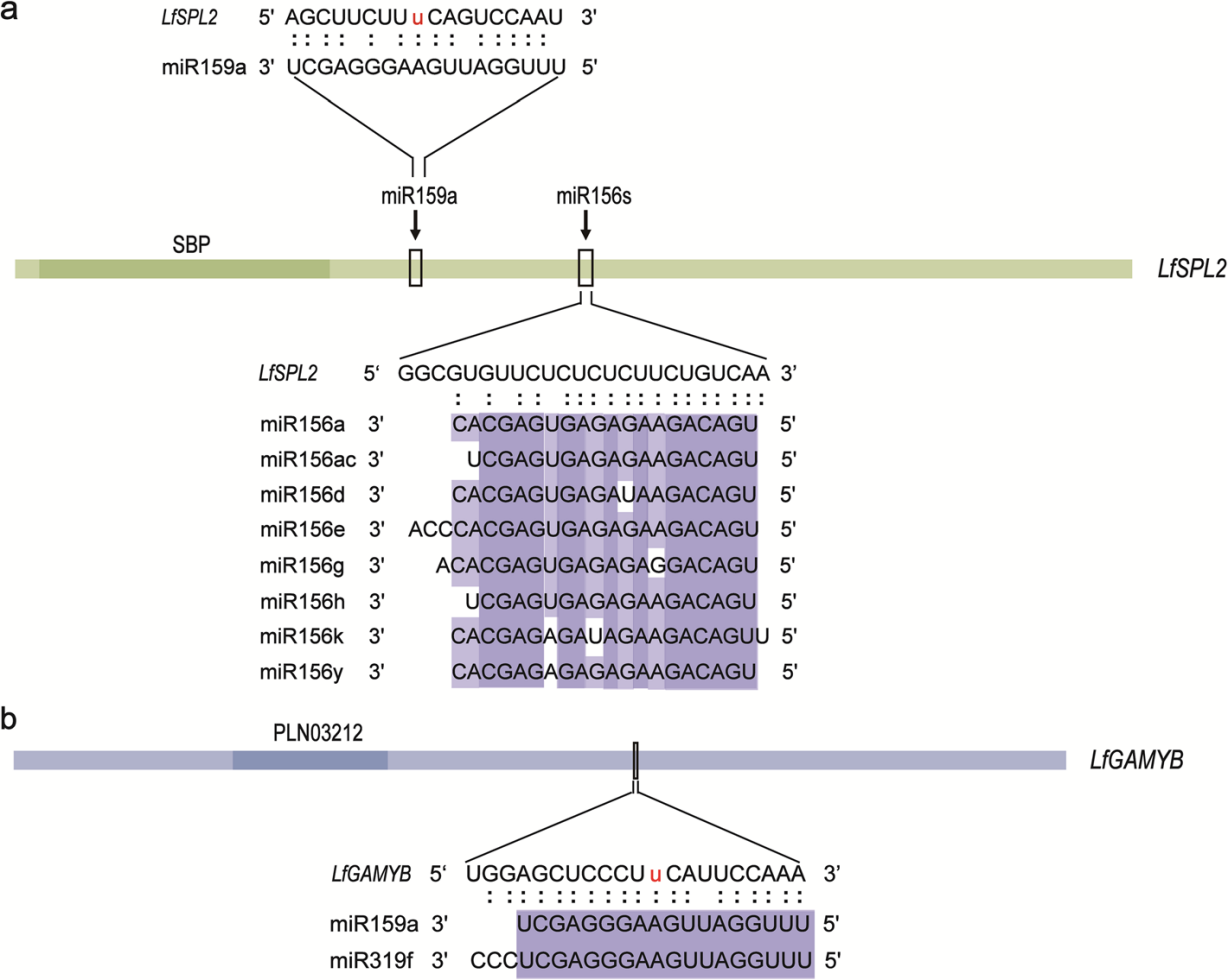
mRNAs simultaneously targeted by miRNAs belonging to multiple families. **a** Diagram of *LfSPL2* and *LfGAMYB*. Conserved domains are included, and the boxes represent the miRNA target sites. **b** Alignment of the miRNA targeting sites of miR156s-*LfSPL2*, miR159a-*LfSPL2* and miR159a/miR319f-*LfGAMYB*

According to the functional annotations, putative flowering-related targets were identified. They consisted of squamosal promoter-binding, floral homeotic protein APETELA2-like, MADS-box, NAC domain-containing, R2R3-MYB, GAMYB and hormone signalling auxin response factor 18-like proteins (Table 2). We also found that the functions of *LfSPL2* and *LfNAM*, targeted by miR156s and miR164a, were enriched in starch and sucrose metabolism, pentose and glucuronate interconversion pathways. Furthermore, both miR156s and miR159a were indicated to regulate the common target *LfSPL2* according to the identification of different target sites (Fig. 5a). miR159a and miR319f targeted *LfGAMYB* sharing a common targeting site (Fig. 5b). These transcripts targeted by miRNAs were considered candidate key regulators during vegetative development and flowering induction in lily.

**Table 2:** Identification of putative flowering-related target transcripts in *L.* × *formolongi*

miR_name	Transcript	Transcript Annotation	Symbol	Term
lfo-miR156a,k,e,ac,d,g,y,r,h	Unigene18229_All	squamosa promoter-binding-like protein 12-like	*LfSPL2*	Starch and sucrose metabolism-
lfo-miR156a,e,ac,d,g,r,y	Unigene9969_All	predicted protein	*LfSPL3*	–
lfo-miR159a,f,c	Unigene2305_All	transcription factor GAMYB	*LfGAMYB*	developmental process
lfo-miR159a	Unigene18229_All	squamosa promoter-binding-like protein 12-like	*LfSPL2*	Starch and sucrose metabolism-
lfo-miR159e	CL730.Contig4_All	transcription factor TCP2-like		–
lfo-miR160a,f,g,b,d	CL6847.Contig1_All	auxin response factor 18-like	*LfARF*	root cap development; auxin mediated signaling
lfo-miR164a	CL6145.Contig1_All	NAC domain-containing protein	*LfNAC*	–
	Unigene9281_All	no apical meristem-related protein	*LfNAM*	Starch and sucrose metabolism; Pentose and glucuronate interconversions;
lfo-miR172a	CL1418.Contig1_All	vesicle-associated membrane protein		response to salt stress
	Unigene18548_All	floral homeotic protein APETALA 2-like	*LfAP2*	–
lfo-miR319f	Unigene2305_All	transcription factor GAMYB	*LfGAMYB*	developmental process
lfo-miR394	Unigene14281_All	F-box family protein		regulation of auxin mediated signaling
lfo-miR828	Unigene3673_All	transcription factor R2R3-MYB		
lfo-miR5179	Unigene7245_All	MADS-box transcription factor	*LfAGL15*	regulation of transcription, DNA-dependent

### Correlation analysis of miRNA expression profiles and miRNA target genes

**Fig. 6:**
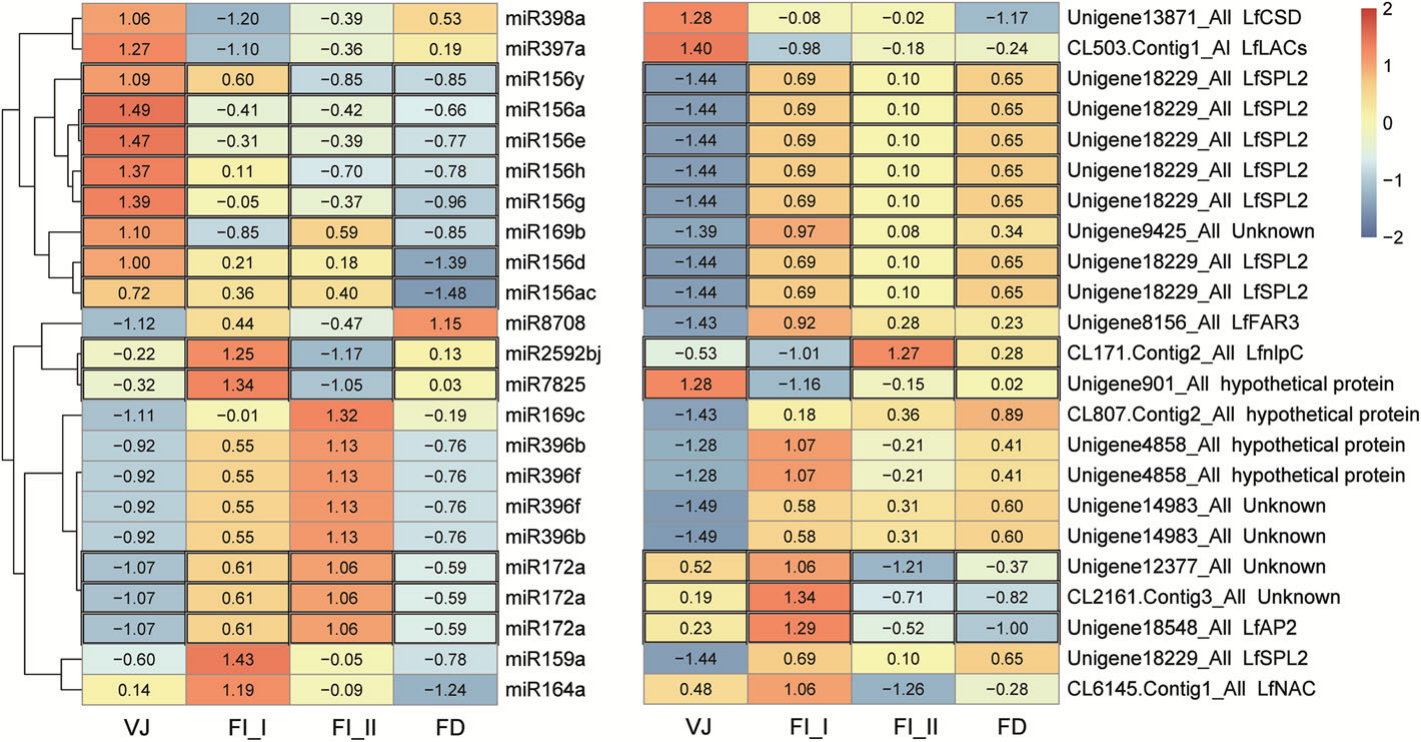
Heat map showing the expression patterns of differentially expressed miRNA-target. These miRNAs targeting differentially expressed mRNAs were clustered by hierarchical clustering at p ≤ 0.05 according to their expression patterns. The black-framed portion represents the expression level of miRNA and mRNA that showed negative correlation relationship at the expression level

To investigate the crucial participants and their variable trends during lily vegetative development and flowering initiation, the 28 detected target transcripts of significantly differentially expressed miRNAs obtained from the degradome were applied for differentially expressed genes (DEGs) analysis. There were 15 DEGs corresponding to 23 differentially expressed miRNAs in the transcriptome from the VJ (vegetative growth) to FD stages (Fig. 6). Among them, 13 miRNA-mRNA pairs displayed negative correlations at expression level. The members of the miR156 family were most highly expressed and displayed significantly downregulated expression levels, while the expression of their target *LfSPL2* increased during development.

**Fig. 7:**
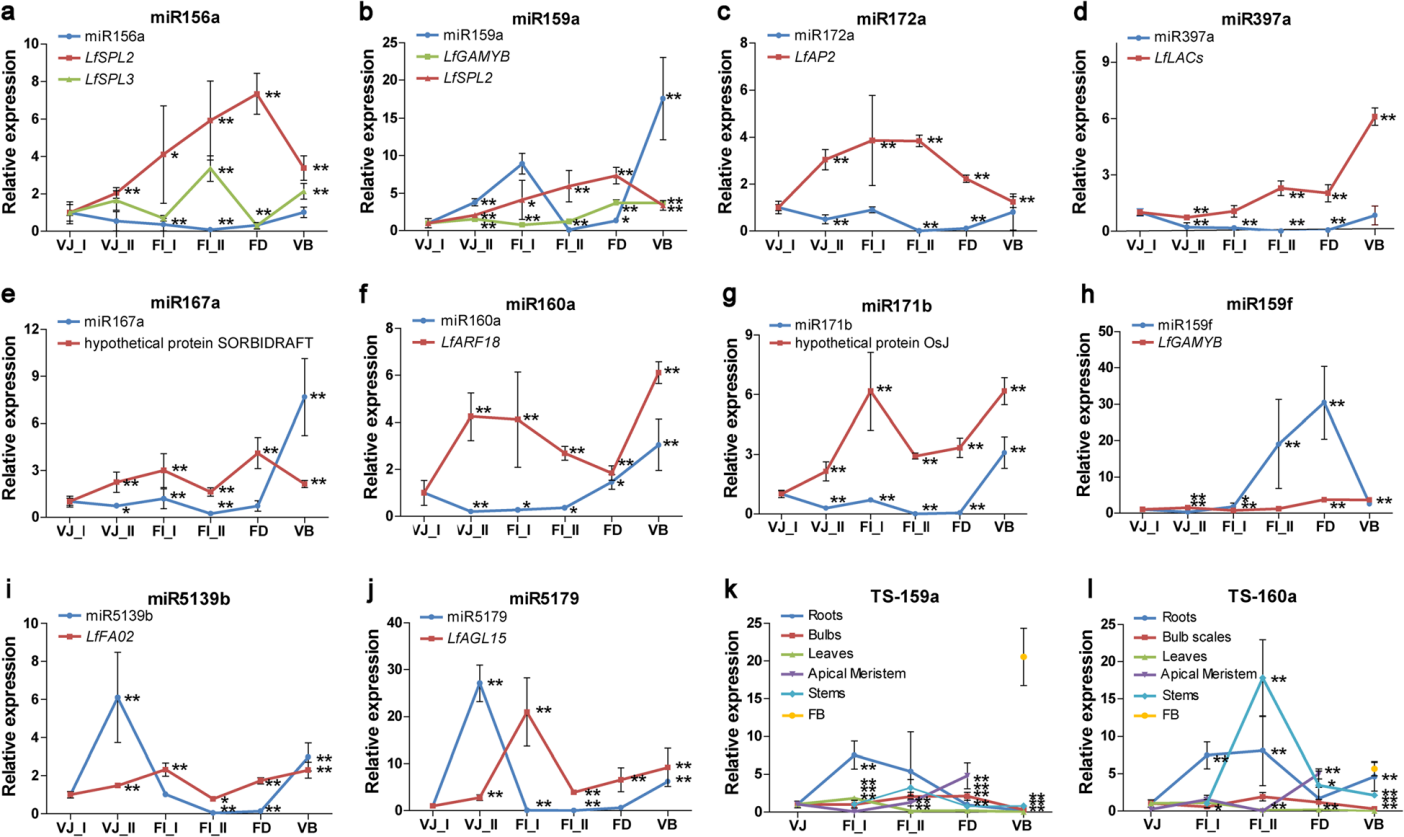
The expression patterns of miRNAs determined by RT-qPCR during development in *L.* × *formolongi*. **a**-**j** The expression patterns of miRNAs and their targets. **k**-**l** The tissue-specific expression profiles of miR159a and miR160a during lily development processes. The normalized miRNA and target levels at the VJ_I stage were arbitrarily set to 1. Each bar represents the mean ± SE of triplicate assays. * or ** indicates a statistically significant difference relative to the value at VJ_ I for each miRNA at *p* < 0.05 or 0.01, respectively

Based on the results of the correlation and functional analyses of the targets, 12 miRNA-target pairs that showed distinct expression patterns and may take part in flowering induction were selected to verify the accuracy of the sequencing results by RT-qPCR analysis. As depicted in Fig. 7a-j, these 10 candidate miRNAs showed substantially similar expression changes to those indicated by the data from the miRNA libraries. A portion of these miRNAs were inversely correlated with their corresponding targets, such as miR156a-*LfSPL2*/ *LfSPL3*, miR159a-*LfGAMYB*, miR397a-*LfLACs*, miR160a-*LfARF18*, miR5139b-*LfFAO2*, and miR5179-*LfAGL15*, which confirmed the negative relationship in the expression profile of miRNA-mRNA modules. Moreover, the expression profiles of the miRNAs from the VJ_I to FD stages were diverse. miR160a was gradually upregulated after the VJ_I stage, while four miRNAs, including miR159a, miR172a, miR167a and miR171b, exhibited upregulated expression at the FI_I stage. Both miR5139b and miR5179 presented expression peaks at the VJ_II stage. All these miRNAs exhibited a remarkable upregulation of transcript abundance from the FD to VB stages, except for miR159f, which exhibited decreased expression at the FD stage.

We also assayed the tissue-specific expression profiles of two significantly differentially expressed miRNAs, miR159a and miR160a (Fig. 7k, l). They showed similar expression patterns in different organs and tissues from the VJ to VB stages. Both presented high expression in roots and stems at the flowering induction (FI_I, FI_II) stages. Expression peaks of miR159a and miR160a were found in leaves at the FI_I stage. However, the level of miR159a increased from the VJ to FD stages in the apical meristem and bulb scales. In visible floral buds, the strikingly highest expression of mR159a was observed. Likewise, miR160a showed the highest expression level at the FD stage in the apical meristem.

### Gene coexpression network analysis

To mine the highly correlated miRNA clusters that were relevant to flowering induction, we used miRNA data obtained from high-throughput sequencing to conduct correlation network analysis by WGCNA. After testing for abnormal samples or genes, correlation networks were constructed by setting the soft threshold to 12 (Additional file 4: Fig. S4–7). As a result, 5 miRNA coexpression modules comprising 70 (blue module), 40 (brown module), 53 (green module), 82 (turquoise module) and 33 (yellow module) interconnected miRNAs were found, as shown in Additional file 2: Table S7. We also recognized 14 miRNAs belonging to differentially expressed miRNA-target pairs in the blue module (miR169b, miR156a/ h/ e/ y), brown module (miR164a, miR172a, miR2592bj), green module (miR160a), turquoise module (miR156d/ g/ ac) and yellow module (miR398a, miR397a). The greatest number of these miRNAs belonging to differentially expressed pairs was found most in the blue module; in this regard, the blue module could be considered the key module related to critical stages of vegetative development and flowering initiation in lily.

**Fig. 8:**
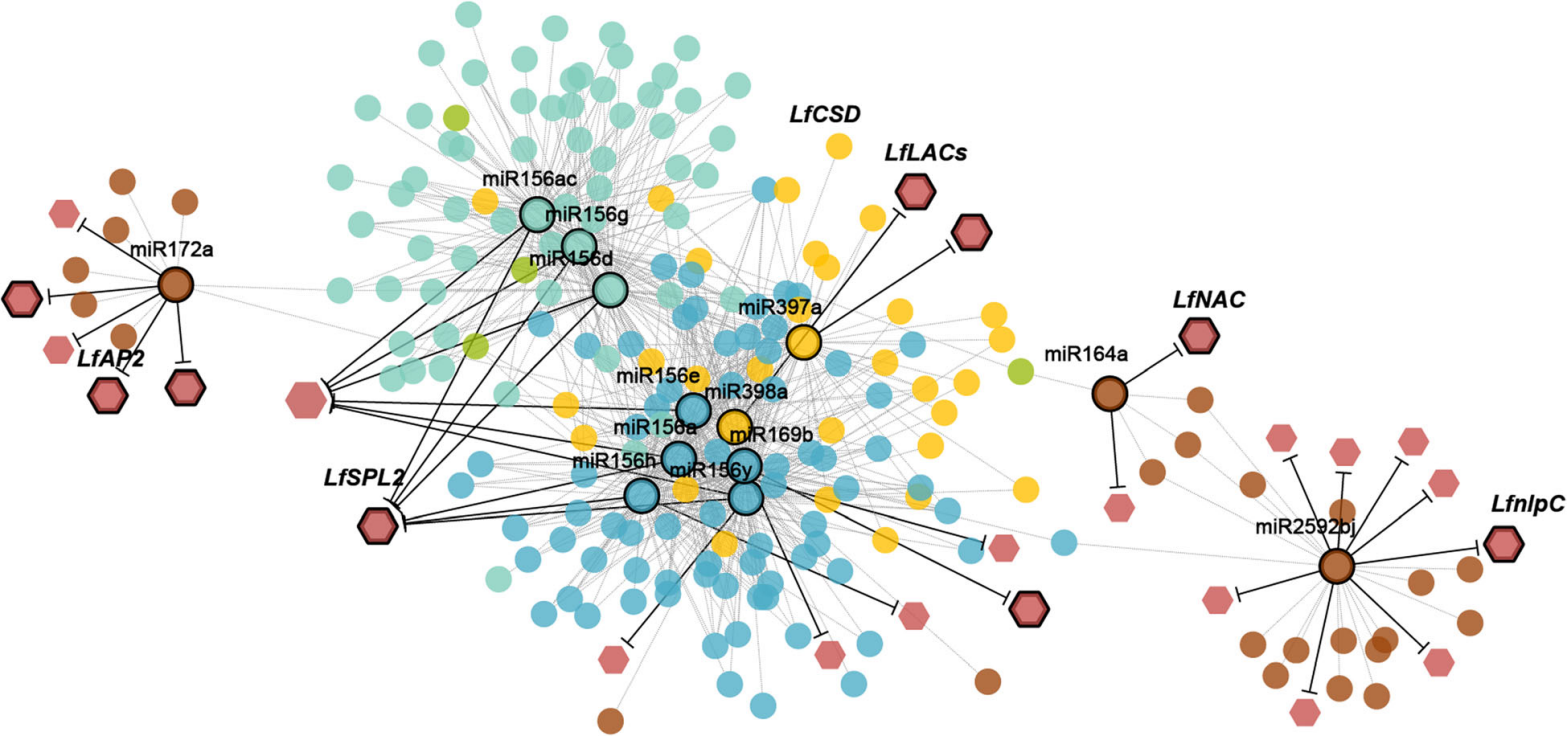
The coexpression subnetwork of putative crucial flowering-related miRNAs and their targets. The coexpression subnetwork of putative crucial flowering-related miRNAs and their targets in lily. In the nodes, circles and diamonds represent miRNAs and target transcripts, respectively. Different colours of nodes represent different modules identified by WGCNA

We also extracted a subnetwork containing 795 edges connected with the differentially expressed miRNAs, which also included their targets (Additional file 2: Table S8). In the regulatory network, miR156a/ d/ e exhibited the greatest numbers of interactions with other sequences, suggesting their core roles among the coexpressed genes (Fig. 8). Moreover, phylogenetic analysis demonstrated that miR156s were most closely related to *Arabidopsis*. They are likely candidate miRNAs involved in lily flowering induction, and the specific roles of the members of this family require further validation and investigation (Additional file 5: Fig. S8).

## Discussion

### Potential crucial miRNAs in vegetative development and flowering induction

The role of miRNAs in flowering induction has been confirmed in different plants, such as *Arabidopsis*, rice, maize, apple and tomato [19, 30, 31]. The mechanism of miRNA functions in the genetic network underlying lily vegetative development and flowering initiation is poorly understood.

In our study, 84 miRNAs showing significant differential expression were identified, and analysis of their expression dynamics during lily development was conducted. The greatest numbers of differentially expressed miRNAs were identified and strongly expressed at the VJ_I and VJ_II stages among the small RNA libraries. These results were consistent with our hypothesis, and we reason that miRNA-mediated modulation is crucial for vegetative development by integrating environmental and endogenous factors to prepare for the induction of flowering.

In the *L.* × *formolongi* libraries, miRNAs belonging to the miR156 family were the most frequent among the differentially expressed miRNAs. In *Arabidopsis*, miR156 is an important orchestrator of the ageing pathway that controls the vegetative-to-reproductive transition and shows very high expression in the vegetative phase [32]. The conserved expression profile of miR156 members has been confirmed in many plants, such as rice [33], maize [34], tobacco [35] and the perennial woody species *Acacia confuse*, *Eucalyptus globulus*, and *Quercus acutissima* [36]. In *L.* × *formolongi*, the miR156 families shared similar expression profiles with those observed in other plants during development, demonstrating their evolutionary and functional conservation and their dominant role during vegetative development. Nevertheless, the expression level of miR156k fluctuated and was upregulated at the FI_I stage. In *Arabidopsis* and rice, *Ath-MIR156A*/ *C* and *OsmiR156b*/ *h* play master roles among miR156 precursors [37–39]. We observed that the precursors of miR156s (*MIR156k*) were phylogenetically closely related to *Arabidopsis* sequences and that *MIR156k* exhibited the closest relationships with *Ath-MIR156A*/ *C*, which clustered into the same branch (Additional file 5: Fig. S8), indicating that miR156k precursors may have a greater effect on the abundance of miR156 during lily flowering induction than during the juvenile-adult phase transition.

A prior study in *Arabidopsis* confirmed that another crucial regulator, miR172, exhibits an inverse expression pattern with miR156 and shows consistently upregulated expression during development to regulate the phase transition as well as floral organ identity [40, 41]. There are five miR172 family members and miR172b could be negatively regulated by miR156e to influence the juvenile-adult phase change [32]. In the perennial species *Jatropha curcas*, only two miR172s have been identified [42]. However, ten miR172s and only one miR172 (miR172a) were identified among the differentially expressed miRNAs in our libraries. The expression of miR172a was elevated from the VJ to FI_II stages, followed by a decline at the FD stage. Hence, we consider miR172a to exhibit relatively complex or species-specific functions in lily flowering induction or other aspects of development.

In *Arabidopsis* and rice, miR159a, miR167a and miR160a affect the development of anthers and sepals [43–45]. In lily, these highly conserved miRNAs also showed significantly upregulated expression at the VB stage, suggesting their important roles in floral organ formation during early floral development. In addition, miR5179 affects the diversification of perianth organs by cleaving MADS-box genes in orchids [46]. In contrast, miR5179 presented upregulated expression at the VB stage but exhibited strikingly higher expression at the VJ stage, indicating that this less conserved miRNA, which is rarely observed in other plants, may exhibit a more crucial function during the vegetative development of lily than during flower development.

### Flowering-related miRNA-mRNA modules in *L.* × *formolongi*

miRNAs carry out their biological functions by regulating the expression of specific mRNAs. We identified targets that encoded flowering-related transcription factors or proteins. *SPL* genes are targeted by miR156 and regulate many aspects of plant vegetative development as important components of multiple pathways [47]. In *Arabidopsis*, 10 *SPL* genes are regulated by miR156, and *AtSPL3*/ *14*/ *15*/ *9* can promote flowering [4, 48]. *SPL7* and *SPL8* participate in flowering modulation and are targeted by miR156 in grasses [49]. Among the 11 *LfSPL* members, two homologs, *LfSPL2* and *LfSPL3*, were targeted by miR156s in *L*. × *formolongi*, and only miR156s-*LfSPL2* pairs were differentially expressed. Moreover, miR156a/ d/ e were located at the cores of the coexpression networks. Accordingly, miR156a/ d/ e-*LfSPL2* modules may be the most critical regulators of the ageing pathway during vegetative development and flowering induction. The mechanism by which the miR156-*LfSPL* module is involved in the genetic network underlying flowering requires further intensive investigation and verification.

Previous studies in *Arabidopsis* have confirmed that miR159 regulates the juvenile-to-adult phase transition through a canonical miR159-*GAMYB*/ *MYB33* interaction [50], and *MYB33* could also activate miR156 as well as the target *SPL9* [17]. Interestingly, we found that miR159a regulated *LfSPL2* simultaneously with miR156s, in addition to *LfGAMYB* in this study. miR159a exhibited high expression levels at the FI_I stage, and the expression level of miR159a and *LfSPL2* exhibited an approximately negative relationship from FI_I to VB stage. Hence, the results imply that miR159 may mainly promote the juvenile-to-adult phase change and flowering induction via the miR159-*LfGAMYB* module. Meanwhile, miR159a might be involved in a new flowering pathway through which it affects flowering initiation by regulating the *SPL* gene directly, and this regulatory module is worthy of validation in the future.

### Crucial factors affecting the vegetative development of lily

A recent study conducted in our laboratory suggested that when the seedlings of *L.* × *formolongi* undergo bolting, the seedlings transition into the reproductive stage, and the juvenile-adult transition is accomplished [3]. Therefore, we focused on the miRNA-mediated regulatory mechanism during flowering induction, particularly during vegetative development at the rosette stage of *L*. × *formolongi*, and the factors affecting this process.

In the molecular regulatory network, sugar acts upstream of miR156 to alter miR156 expression [37]. T6P influences flowering in the leaves and apical meristem by regulating *FT* as a representative of carbohydrate conditions [6]. Previous studies have shown that daylength strongly affects the bolting time and quality of *L.* × *formolongi* [25]. Temperature also plays an important role in vegetative development. Cold exposure induces flowering by breaking bulb dormancy and influences the growth of bulbous plant species [51]. Our study showed that the functions of the identified targets were enriched in cadmium ion, cold response and sugar metabolism pathways, including the pentose phosphate pathway and carbon fixation in photosynthetic organisms. Thus, vegetative development may be closely associated with carbohydrates, cold or photoperiod in *L.* × *formolongi* via miRNAs. *LfSPL2* and *LfNAM*, which are targeted by miR156s and miR164a, respectively, were identified in the sugar metabolism pathway. Hence, the impacts of these factors on the vegetative development of *L.* × *formolongi* through the potential miRNA-mRNA modules is also an important topic awaiting future exploration.

**Fig. 9:**
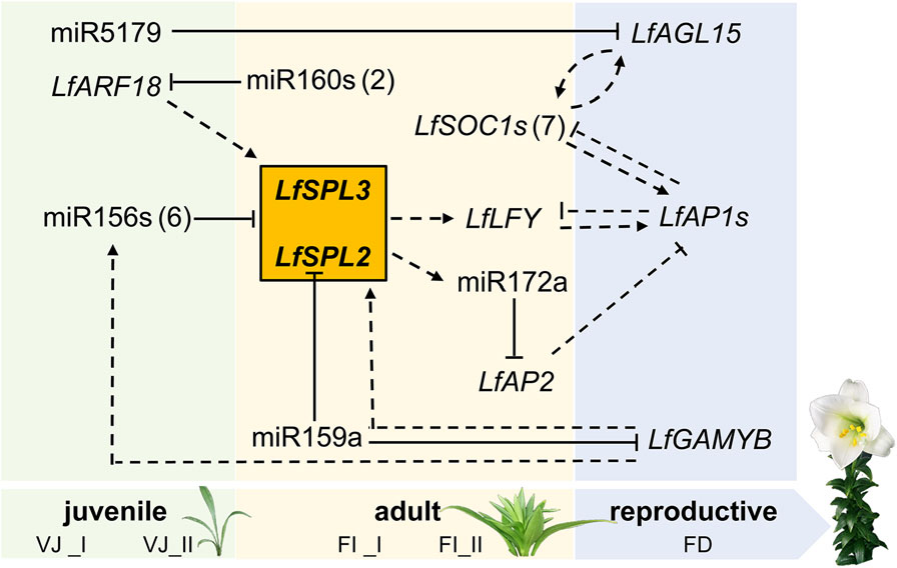
The putative miRNA-mediated molecular regulation network of flowering induction and development of *L.* × *formolongi*. The locations of the genes are determined by their expression patterns. Genes in green background displayed expression peak at the VJ stage, genes in the yellow and blue background displayed up-regulated expression at the FI stage and FD stage respectively. The numbers of differentially expressed genes are also presented. Arrows in dotted lines represents the potential regulatory relationship between two genes

Taking the flowering regulation network in Arabidopsis as a reference, the hypothetical schematic diagram was based on the analysis of the small RNA libraries, degradome and transcriptome data (Fig. 9). In Arabidopsis, miR156 and *SPLs* are master regulators in vegetative development phase transition. SPL proteins can induce floral integrators, *SUPPRESSOR OF OVEREXPRESSION OF CONSTANS 1* (*SOC1*) and *AGL42* in leaves and apical meristem [13]. In apical meristem, *SPLs* can also regulate the floral meristem identity genes *LEAFY* (*LFY*) and *APETALA 1* (*AP1*), which are repressed by *AP2* transcription factor genes, the target of miR172 [13, 15]. Meanwhile, *SPL9* directly promotes the transcription of miR172 to affect vegetative-to-reproductive phase transition [13]. Additionally, *ARF 3*/*4* may modulate the expression of *SPL3* via regulating the juvenile-adult phase transition [52].

Similar to Arabidopsis, the miRNA-target pair miR156-*SPL* occupies a central position in the putative miRNA-mediated flowering regulation network in *L*. × *formolongi*. The decreased expression of miR156 induces *LfSPL2* and *LfSPL3* expression at the FI_II stage, which may promote the MAD-box gene *LfLFY* to induce flowering in apical meristem. Meanwhile, miR160s could target *LfARF18*, and up-regulated *LfARF18* at the VJ_II stage might promote the expression of *LfSPL2* and *LfSPL3*. miR159a might be involved in flowering induction and floral meristem formation via repressing the expression of *LfGAMYB* and *LfSPL2*, respectively. Additionally, *LfGAMYB* may also induce the regulators miR156s and *LfSPLs* in the ageing pathway. *LfSPL2* and *LfSPL3* may also activate miR172a transcription. miR172a targets *LfAP2*, and the down-regulated expression of *LfAP2* before VB stage may repress *LfAP1* and *LfSOC1*. In addition, miR5179 specifically target MADS-box gene *LfAGL15*, which may play critical roles at flowering induction stage.

## Conclusions

To our knowledge, the present study provides the first integrated transcriptomic, miRNA library and degradome analysis conducted to obtain comprehensive knowledge of miRNAs and their targets during flowering initiation in lily. Crucial miRNAs and miRNA-target pairs involved in vegetative development and floral induction in *L*. × *formolongi* were identified via the correlation analysis of sequencing datasets. Factors including carbohydrates, cold and photoperiod and associated enriched target functions may affect vegetative development and flowering induction. These results will help us to gain broader knowledge of the miRNA-mediated mechanism modulating *L*. × *formolongi* vegetative development and flowering and will be of great importance for regulating growth and development processes in lily.

## Methods

### Plant materials and samples collection

The seeds of *L.* × *formolongi* (‘Razan No. 2’) (purchased from Murakami Seed, Ibaraki, Japan) were sown after stratification at 4 °C for one month. Seedlings with 2–3 rosette leaves were transplanted into individual pots and grown in a chamber under a 16 h light (25 °C)/ 8 h dark cycle (16 °C) with 70% humidity, illuminated by white light with a 320 μmol·m^− 2^·s^− 1^ photosynthetic photon flux density.

A previous study in our laboratory confirmed the key characteristics of vegetative development and flowering initiation in *L.* × *formolongi* under long-day conditions through morphological and gene expression pattern investigations [3]. Therefore, fresh leaves and apical stems were collected for small RNA library construction during different developmental stages, including the 2–3 rosette leaf stage (vegetative juvenile_I, VJ_I), 5–6 rosette leaf stage (vegetative juvenile_II, VJ_II), 1–2 internode stage (flowering induction_I, FI_I), 5–6 internode stage (flowering induction_II, FI_II) and 9–10 internode stage (floral differentiation, FD). Similar samples from these stages together with fresh leaves and buds collected from 70 to 71 internodes (visible floral bud stage, VB) were prepared for RT-qPCR analysis. To examine the tissue-specific expression patterns of the differentially expressed miRNAs miR159a and miR160a, fresh leaves, apical meristems, middle stems, bulb scales (in the middle of the bulb), roots and tiny floral bubs (approximately 2 cm) were also collected from one plant from the VJ_I to VB stages. Three biological replicates were collected for each developmental harvesting point. All samples were immediately frozen in liquid nitrogen, followed by storage at − 80 °C for further study.

### Small RNA library construction and sequencing

Total RNA was extracted using the EASY spin Plus Plant RNA kit (Aidlab Bio, Beijing, China) according to the manufacturer’s instructions. The high-quality total RNA was used for small RNA libraries generation following the TruSeq Small RNA Sample Prep Kits protocol (Illumina, San Diego, USA). Briefly, after ligating the RNA with 3′ and 5′ adapters, RNAs were used for reverse transcription and PCR amplification. Then gel electrophoresis was performed and gel-purified small RNA libraries of *L.* × *formolongi* at different development and flowering initiation stages were constructed followed by quality control analysis. Sequencing was conducted with Illumina HiSeq 2500 platform (LC Bio, Hangzhou, China). Each reaction was performed in triplicate.

### Bioinformatic analysis of sRNA data

The sequencing analysis were performed through an in-house program, ACGT101-miR (LC Bio, Hangzhou, China). Briefly, to obtain valid reads without a reference for the *L.* × *formolongi*, sequence adapters, low-quality reads and junk reads containing poly-N were filtered out. Subsequently, the filtered reads that matched noncoding RNAs (rRNAs, tRNAs, snRNAs and snoRNA) and repeats in RFam (http://rfam.xfam.org) and Repbase (http://www.girinst.org/education/index.html) databases were discarded. The remaining 18–25 nt valid reads were further processed to summarize the length distribution, and mapping to specific species precursors in miRBase 22.0 via BLAST searches and transcriptome (data not published) analysis of *L.* × *formolongi* were performed to identify known miRNAs. The miRNAs are categorized into 3 groups, including known miRNAs (perfectly matched sequences, group 1) and conserved miRNAs (group 2 and group 3), according to whether the reads and precursors could be matched to sequences in miRBase and the transcriptome. Among the conserved miRNAs, the reads could be aligned to precursors in miRbase and the mapped precursors failed to be mapped to the transcriptome. When the reads could also be mapped to the transcriptome, the reads were categorized in group 2, if not, they were categorized in group 3.

After extracting the known miRNAs, the valid reads that were unmapped in miRBase but were subjected to BLAST searches against the transcriptome of *L.* × *formolongi* were selected for novel miRNA identification. RNAfold software (http://rna.tbi.univie.ac.at/cgibin/RNAWebSuite/RNAfold.cgi) was employed to predict the secondary structures of potential miRNA sequences. The detailed criteria applied were as follows: (1) numbers of nucleotides in a single bulge in the stem and the mature region were ≤ 12 and 4, respectively; (2) in a predicted hairpin, number of base pairs in the stem region was ≥16; (3) in the mature region, both numbers of biased errors and bulges in one bulge and the number of errors were ≤ 2 [53].

### Analysis of differentially expressed miRNAs

To identify the differentially expressed miRNAs in *L.* × *formolongi* during the flowering transition phase and development, the deep-sequencing counts were normalized as described in prior reports [54]. In brief, first, a reference data set made up of the copy number median of selected common sequences of all samples was constructed. Second, the copy numbers of total samples from all libraries were transformed into Log2 (copy#) followed by calculation of differences between individuals and reference data set. Third, the linear regressions between the samples and reference set on the subset sequences (|△Log2 (copy#)| < 2), and the arithmetic correction factor were generated to calculate the expected Log2 (copy#). Thus, we calculated the expected Log2 (copy#) values to indicate the original copy numbers of the samples. The differentially expressed miRNAs were identified by one-way ANOVA and *p*-values were obtained via multiple comparisons between the libraries. The miRNAs with p-values ≤0.05 in each test were defined as significantly differentially expressed miRNAs during lily development.

### Degradome library construction and target identification

Total RNAs from the fresh leaves and apical meristem of *L.* × *formolongi* at five developmental stages were equally mixed as a single sample to construct the degradome library according to published protocols [55]. Degradome cDNA library sequencing was also processed using Illumina Hiseq 2500 (LC Bio, Hangzhou, China). The raw reads were processed to remove adaptor sequences and low-quality sequences, the clean reads were generated and subjected to potential cleaved target identification by comparison with transcriptome sequences according to the description in previous study [56]. Thus, truncated transcripts resulting from endonucleolytic cleavage guided by miRNAs could be identified [57]. Furthermore, the alignment analysis of comparisons between identified miRNAs and mapped mRNAs was simultaneously performed using computational target prediction algorithms in TargetFinder (https://github.com/carringtonlab/TargetFinder) software to identify miRNA binding sites. Further annotation of targets was conducted in the Gene Ontology (GO) and Kyoto Encyclopedia of Genes and Genomes (KEGG) pathway databases for further miRNA-gene regulatory network analysis after all target unigenes were subjected to Blast2GO (https://www.blast2go.com/) analysis and aligned against the enriched KEGG pathways following a previously reported method [58].

### Quantitative real-time PCR analysis

Total RNA and miRNA were extracted from leaves and apical meristems at six developmental stages from the seedling to visible floral bud phases under long-day conditions using RNA extraction kits (Aidlab Biotechnology Co., LTD., China; Tiangen Bio, Beijing, China) and miRcute miRNA Isolation kits (Tiangen Bio, Beijing, China) according to the manufacturer’s instructions. The mature miRNA was polyadenylated using the *E. coli* Poly (A) Poly Polymerase Kit (Invitrogen, Carlsbad, CA, USA) [59]. Then, the obtained RNAs and miRNAs were subjected to quality characterization, poly (A)-enrichment and reverse transcription to obtain cDNA using Quant Script RT Kit (Tiangen Bio, Beijing, China) and ReverTra Ace qPCR RT Master Mix with gDNA Remover (Toyobo, Shanghai, China). In addition, to assess tissue-specific miRNA expression, total miRNAs were separately extracted from different tissues from seedlings in the same developmental phases. RT-qPCR was carried out by using THUNDERBRIRD SYBR qPCR Mix Without Rox (Toyobo, Shanghai, China) on a Bio-Rad CFX96 system (CFX96 Touch, BIO-RAD, USA). Each reaction was performed in triplicate. The 5S RNA and *EF* genes were used as the internal reference genes for the normalization of the results [60, 61]. The primers for the miRNA and targets were designed using Beacon Designer 7 software, as indicated in Supplementary Table 6. The − 2^−∆∆Ct^ method was employed to statistically analyse these data [62].

### Coexpression network construction

To generate a correlation network of miRNAs during lily development, an undirected weighted gene network was constructed with a weighted gene coexpression network analysis (WGCNA) algorithm. A tutorial for the WGCNA package in R is provided at https://horvath.genetics.ucla.edu/html/CoexpressionNetwork/Rpackages/WGCNA. The normalized miRNA expression data from the libraries were used for correlation network construction after gene screening with a power of 12. We focused the analysis on module eigengenes whose targets were also significantly differentially expressed during lily flowering. The correlation network of interest was selected for visualization in Cytoscape (version 3.6.1). Moreover, the alignment of the sequences of miRNA precursors and the phylogenetic analysis of miR156 families were performed via the ClustalW and NJ methods in MEGA 6.06 software with default parameters. The precursor sequences of other species were obtained from miRBase, Release 21 (http://www.mirbase.org/index.shtml).

## Supplementary Information

**Additional file 1: Fig. S1**. Pearson correlation between samples. **Fig. S2**. Principal component analysis based on the expression levels in libraries.

**Additional file 2: Table S1**. Conserved miRNA families in lily. **Table S2**. Novel miRNA families in lily. **Table S3**. Significant differential expression of miRNAs in lily. **Table S4**. Identified and annotated target transcripts for the conservative miRNAs in lily. **Table S5**. Identified and annotated target transcripts for the novel miRNAs in lily. **Table S6**. Specific RT-qPCR primers of lily. **Table S7**. The nodes of the gene network weight analysis results generated by WGCNA. **Table S8**. The edges of the gene network weight analysis results generated by WGCNA.

**Additional file 3: Fig. S3**. Predicted hairpin structures of precursors of novel miRNAs. The red-coloured sequences represent mature miRNAs, and the yellow-coloured sequences represent miRNAs

**Additional file 4: Fig. S4**. The selection of soft-thresholding power for the coexpression network construction by WGCNA. 12 was selected as soft- thresholding power parameters. **Fig. S5**. Hierarchical clustering tree. Hierarchical clustering tree showing coexpression modules detected by WGCNA. The major tree branches constitute 5 modules labelled by different colours, including blue, turquoise, yellow, green and brown module. **Fig. S6**. Cluster tree based of the module eigengenes. **Fig. S7**. The correlation coefficient heatmap of the coexpression module genes. Each bright spot corresponds to the correlation between each miRNA and other miRNAs. The deeper the colours, the stronger the connectivity between the two miRNAs in the corresponding row and column.

**Additional file 5: Fig. S8**. The phylogenetic relationship of miR156 family homologs. Neighbor-joining (NJ) tree constructed using precursor miRNA family sequences from *Arabidopsis* (ath), *Oryza sativa* (osa) and *L.* × *formolongi* (lfo). Red dots represent precursor sequences from lily, green dots and blue dots represent precursor sequences from the members which were verified important role in regulating flowering time in *Arabidopsis* and *Oryza sativa*, respectively.

## Data Availability

The sequencing data have been submitted to NCBI SRA (Sequence Read Archive, http://www.ncbi.nlm.nih.gov/sra/), under the accession number PRJNA712953.

## Abbreviations

miRNAs: MicroRNAs
*FT*: *FLOWERING LOCUS T*
T6P: Trehalose-6-phosphate
nt: Nucleotide
*SPL*: *SQUAMOSA PROMOTER BINDING PROTEIN-LIKE*
*AP2*: *APETALA 2*
GA: Gibberellin
*ARF*: *AUXIN RESPONSE FACTORS*
GO: Gene Ontology
KEGG: Kyoto Encyclopedia of Genes and Genomes
NJ: Neighbor-Joining
RT-qPCR: Quantitative real time polymerase chain reaction
WGCNA: weighted gene coexpression network analysis
rRNA: Ribosomal RNA
tRNA: Transfer ribonucleic acid
snoRNA: Small nucleolar RNA
snRNA: Small nuclear RNA
sRNA: Small RNA
mRNA: Messenger RNA
*NAC* (*NAM*): *NO APICAL MERISTEM*
DEGs: Differentially expressed genes
*LAC*: *LACCASE*
*FAO*: *FATTY ALCOHOL OXIDASE*
*AGL*: *AGAMOUS-LIKE*
*SOC1*: *SUPPRESSOR OF OVEREXPRESSION OF CONSTANS 1*
