# Supplementary material for: Analysis of miRNA-mediated regulation of flowering induction in Lilium × formolongi
Source: BMC Plant Biol. 2021 Apr 20;21:190. doi: 10.1186/s12870-021-02961-3 (PMC8058995; doi:10.1186/s12870-021-02961-3)
Supplement: Supplementary file 1 — Additional file 1: Fig. S1. Pearson correlation between samples. Fig. S2. Principal component analysis based on the expression levels in libraries. [file 12870_2021_2961_MOESM1_ESM.docx]

Repeatability analysis between samples


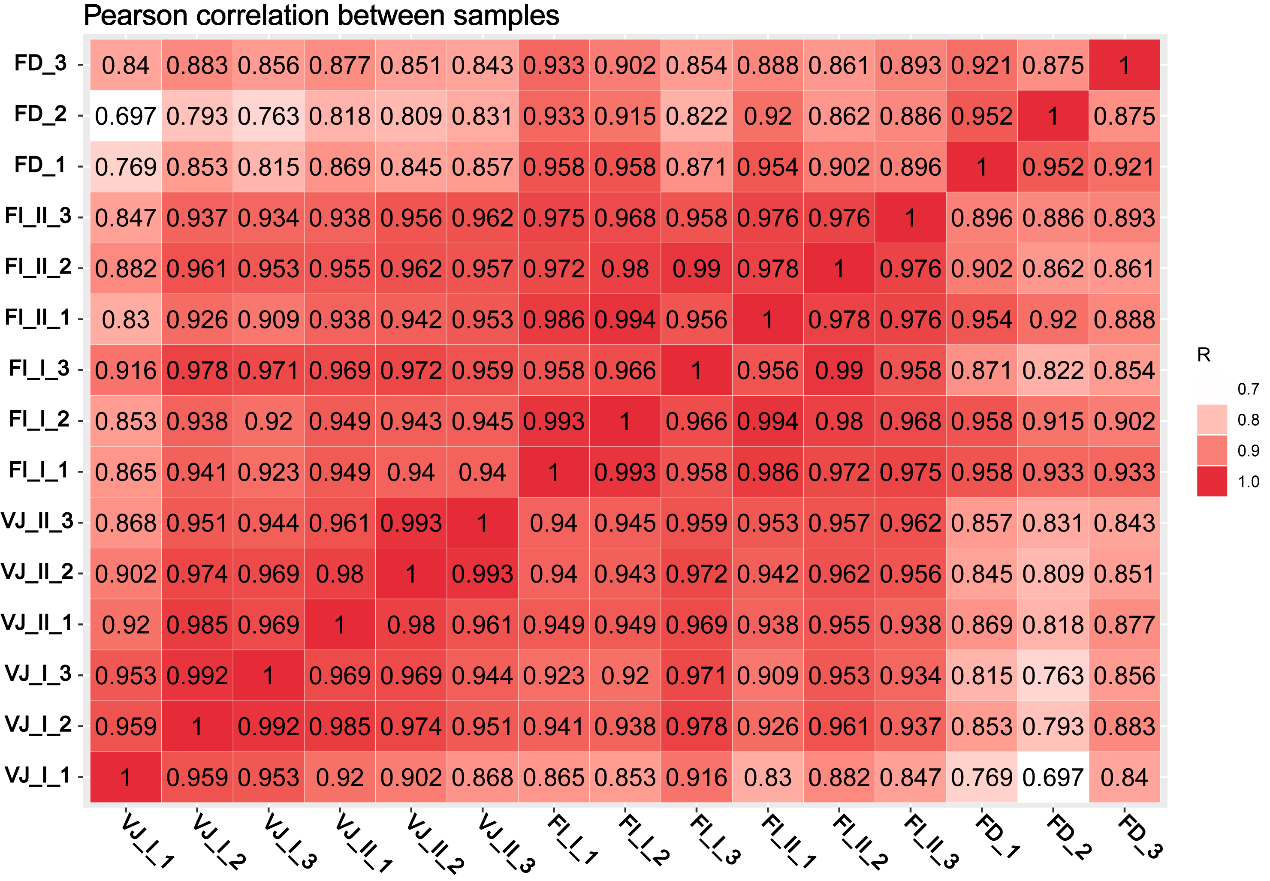


**Fig. S1** Pearson correlation between samples.


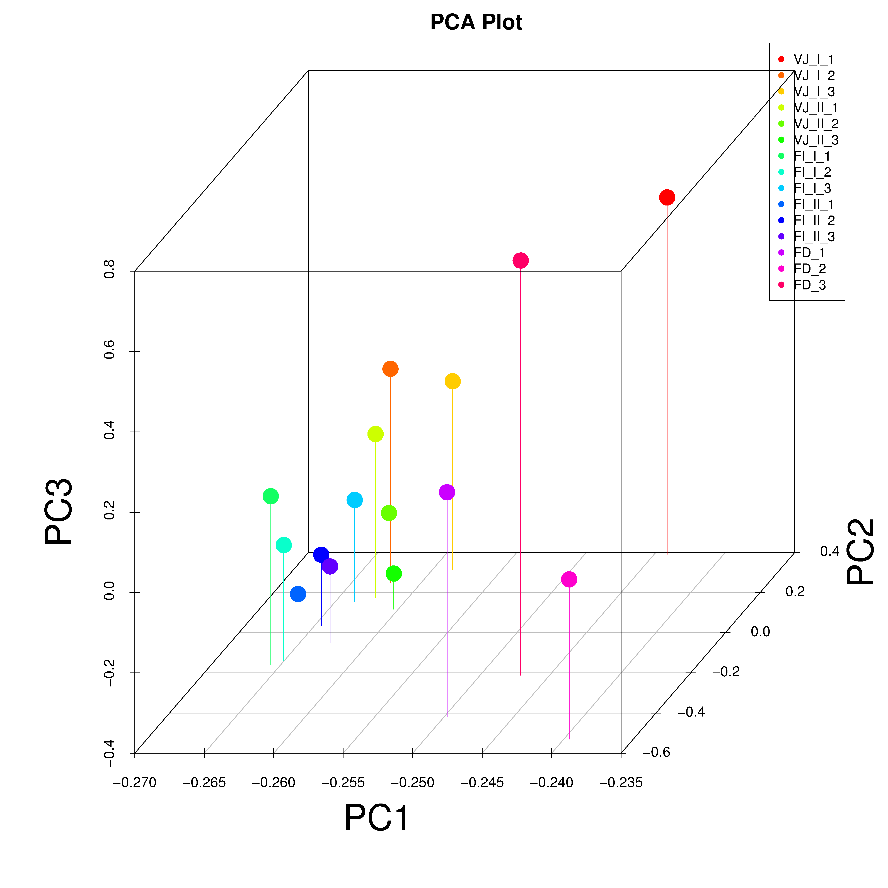


**Fig. S2** Principal component analysis based on the expression level in libraries.
