# Supplementary figures and images for: Analysis of miRNA-mediated regulation of flowering induction in Lilium × formolongi
Source: BMC Plant Biol. 2021 Apr 20;21:190. doi: 10.1186/s12870-021-02961-3 (PMC8058995; doi:10.1186/s12870-021-02961-3)

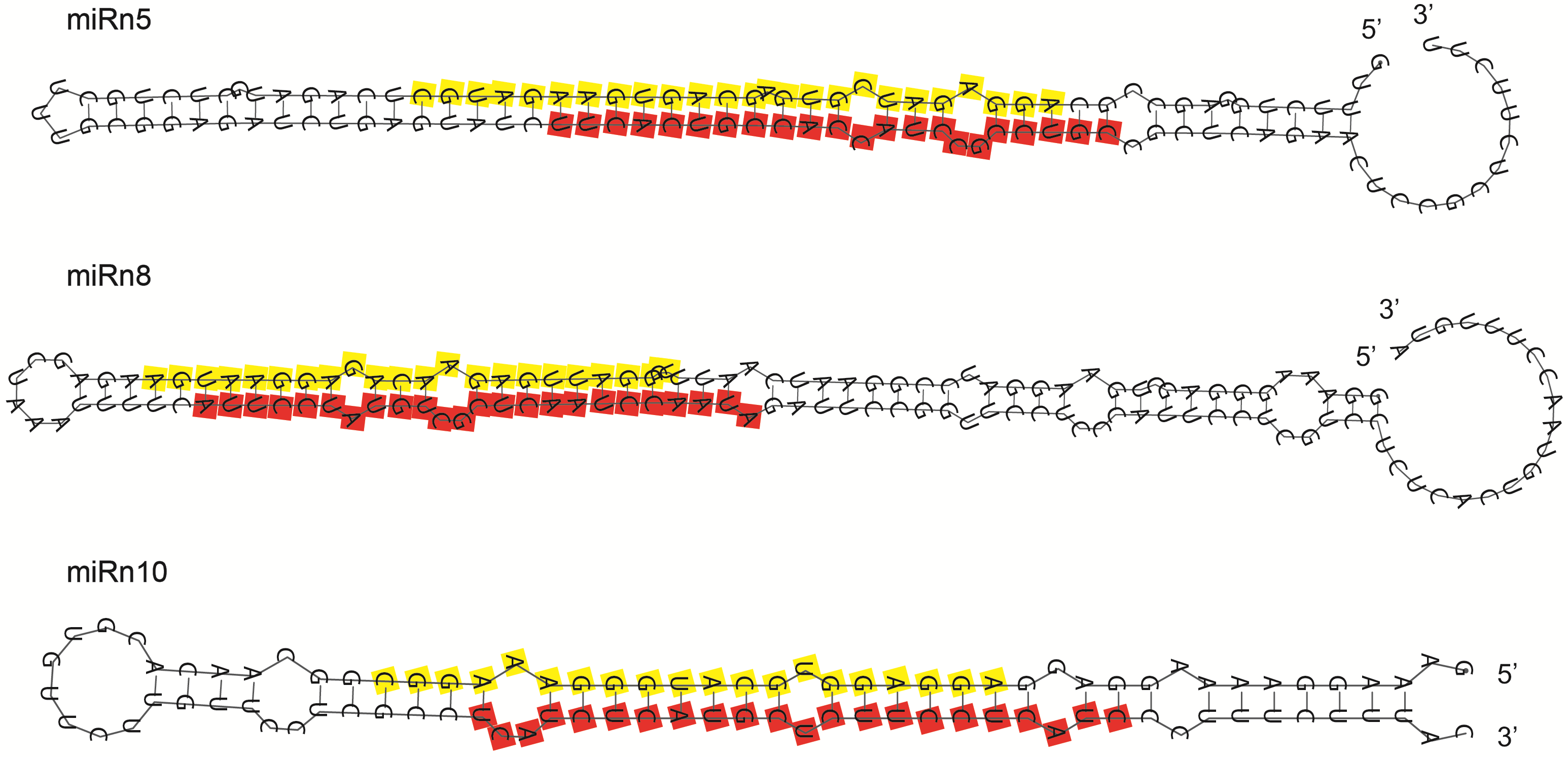

Supplement: Supplementary file 3 — Additional file 3: Fig. S3. Predicted hairpin structures of precursors of novel miRNAs. The red-coloured sequences represent mature miRNAs, and the yellow-coloured sequences represent miRNAs [file 12870_2021_2961_MOESM3_ESM.tif]

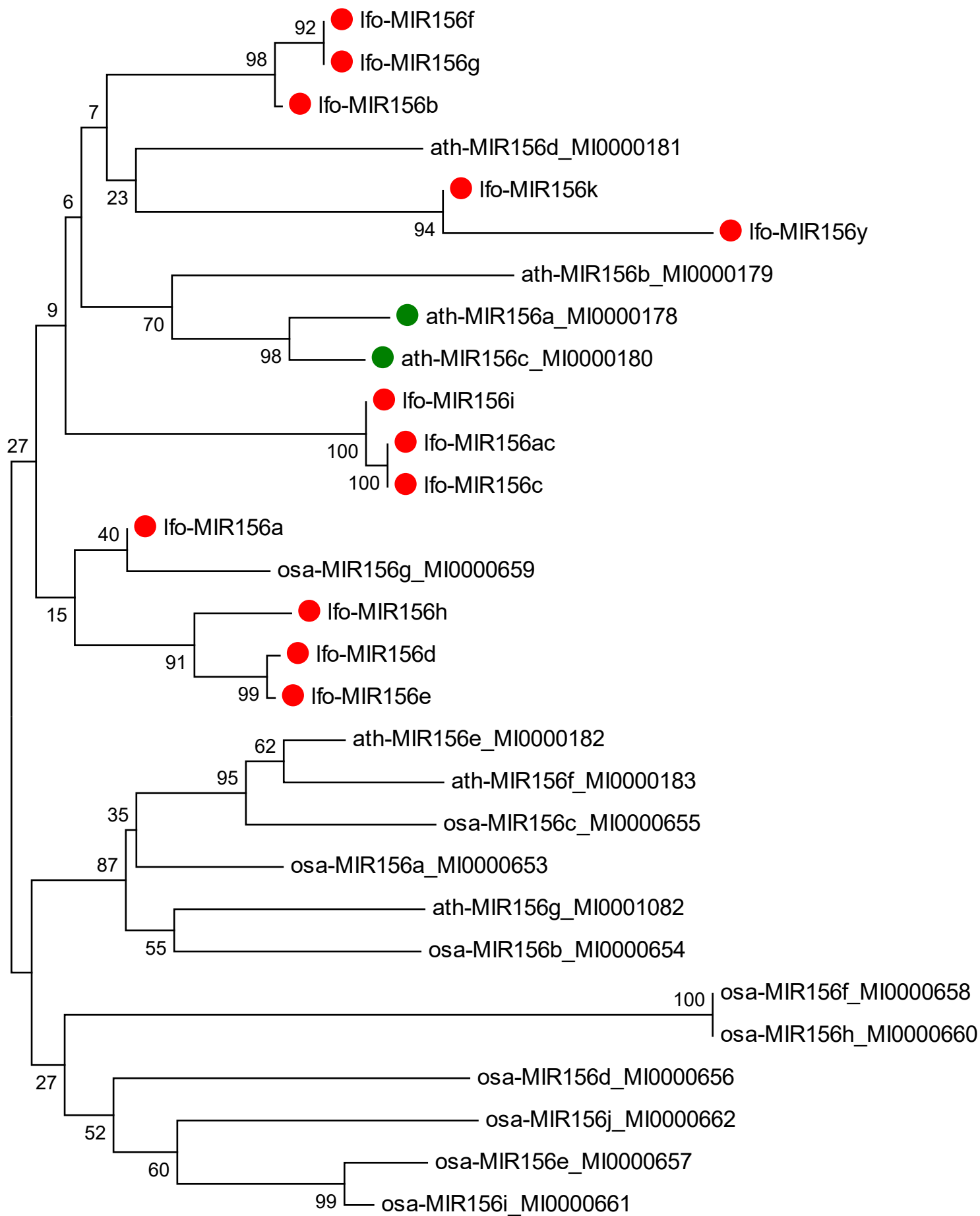

0.05

Supplement: Supplementary file 5 — Additional file 5: Fig. S8. The phylogenetic relationship of miR156 family homologs. Neighbor-joining (NJ) tree constructed using precursor miRNA family sequences from Arabidopsis (ath), Oryza sativa (osa) and L. × formolongi (lfo). Red dots represent precursor sequences from lily, green dots and blue dots represent precursor sequences from the members which were verified important role in regulating flowering time in Arabidopsis and Oryza sativa, respectively. [file 12870_2021_2961_MOESM5_ESM.pdf]
