# Supplementary material for: Analysis of miRNA-mediated regulation of flowering induction in Lilium × formolongi
Source: BMC Plant Biol. 2021 Apr 20;21:190. doi: 10.1186/s12870-021-02961-3 (PMC8058995; doi:10.1186/s12870-021-02961-3)
Supplement: Supplementary file 4 — Additional file 4: Fig. S4. The selection of soft-thresholding power for the coexpression network construction by WGCNA. 12 was selected as soft- thresholding power parameters. Fig. S5. Hierarchical clustering tree. Hierarchical clustering tree showing coexpression modules detected by WGCNA. The major tree branches constitute 5 modules labelled by different colours, including blue, turquoise, yellow, green and brown module. Fig. S6. Cluster tree based of the module eigengenes. Fig. S7. The correlation coefficient heatmap of the coexpression module genes. Each bright spot corresponds to the correlation between each miRNA and other miRNAs. The deeper the colours, the stronger the connectivity between the two miRNAs in the corresponding row and column. [file 12870_2021_2961_MOESM4_ESM.docx]

Coexpression network construction by WGCNA

**Fig. S4** The selection of soft-thresholding power for coexpression network construction by WGCNA. 12 was selected as soft- thresholding power parameters

**Fig. S5** Hierarchical clustering tree. Hierarchical clustering tree showing coexpression modules detected by WGCNA. The major tree branches constitute 5 modules labeled by different colors including blue, turquoise, yellow, green, brown module.

**Fig. S6** Cluster tree based of the module eigengenes.

**Fig. S7** The correlation coefficient heatmap of the coexpression module genes. Each bright spot corresponds to the correlation between each miRNA and other miRNAs. The deeper the colors, the stronger is the connectivity between the two miRNAs in the corresponding row and column.
